# Clinical Reflections

**Published:** 1882-08

**Authors:** Ad. Lippe

**Affiliations:** Philadelphia


### CLINICAL REFLECTIONS: A CONFIRMED SYMPTOM.

AD. LIPPE, M. D., PHILADELPHIA.

A lady, 40 years of age, suffering from what is called "Genuine Contracting Kidney," had improved from time to time under the effects of various remedies. When such improvement secured her good sleep, she always awoke with an irresistible desire to urinate, and then had great difficulty in passing the urine. Cantharides would relieve for a time, but other symptoms, not coming under the pathogenesis of this drug, would supervene, other remedies would again relieve her, and as soon as her sleep was restored, the above symptom would again return. Bronchial catarrh and palpitation of the heart (enlargement of the left ventricle) often became prominent symptoms. Finally, a new symptom became prominent. Pains in the lower vertebræ, as if a hot iron were thrusted through them. This new symptom has been repeatedly confirmed as belonging characteristically to Alumina, and is found in Hahnemann's *Chronic Diseases,* under Symptom 831. In studying out this protractedly tedious case, there was found present also this symptom under Alumina (Symptom 636): "*In the morning, when waking, desire to pass urine with difficult and tardy emission of the urine in a feeble (thin) stream from the female urethra.*" The two next recorded symptoms (637 and 638) had at times been also very prominent in this case. She must rise frequently at night to urinate, with much pale urine. One dose of Alumina (CM Fk.) was administered, and the improvement of the condition of the patient was quite astonishing; it now continues for almost a month, and ever since Alumina was taken there are no nightly calls to urinate, and when she awakens in the morning, the now much more normal urine is voided freely, as if she were in perfect health.

*Comments.*—Attention is called to a not frequently observed symptom, a symptom not necessarily or especially belonging to any form of disease, certainly not necessarily belonging to the genuine contracting kidney, and a symptom which, nevertheless, was an unerring guide to the selection of the truly homœopathic remedy. The pathological condition of the patient had nothing to do with the selection of the remedy. We must again express our detestation of the growing departures from Hahnemann's methods, from the too frequently expressed opinion of labor-fearing men, of men whose only aim is to invent labor-saving methods, who demand that we must first diagnosticate a disease and then find in our materia medica a remedy which has and is capable of causing just such a disease, in other words, press our materia medica into a pathological livery. These same labor-abhorring progressists backwards into the easy allopathic methods, see no use of such a symptom as we hereby report as a *confirmed* symptom.* There are at this day, members of the profession, who claim to be homœopaths, and who, at the same time, wildly applaud the wildest plans to abridge or reconstruct our own materia medica, pronounced by such fablers to be as unscientific as is the materia medica of the old school. *If* such growlers will only go to work and give us one single, newly well-proved remedy, we might see for ourselves what all their talk amounts to. *If* these unfortunate men, seeking what *they* term *positive* symptoms, found them, *if* they then would condescend to ILLUSTRATE the superior usefulness of their *positive* symptoms over the so very-carefully-collected and arranged provings of Hahnemann and a Hering, they would blush of having said, "*and yet we are cherishing false facts enough in our materia medica, to seriously hinder, if not effectually defeat, the application of our great therapeutic law!*" The more we know of our materia medica, the more successfully can we apply our great therapeutic law, and the growler, who cannot apply our materia medica as it is for that purpose, testifies against himself, and blatantly flourishes his utter ignorance of our materia medica and our healing art. Let him, who doubts the correctness of Hahnemann's great work, his *Materia Medica,* do exactly as did the Vienna provers who doubted, seriously, the correctness of Hahnemann's *Materia Medica,* and believed it contained false facts. What did they do? We give docu-

* "Hahnemannian Monthly," May, 1882, page 282.

mentary evidence, to be found in the journals of that day, they *reproved* remedies thirty-five years ago; reproved them in potencies; reproved *Natrum mur.* in the 30th potency, *and* not only confirmed what Hahnemann had published, but greatly added to our knowledge of the pathogenesis of Natrum mur., and the Vienna provers having failed utterly in their attempt to bring discredit on *the Master*, were honest enough to acknowledge their conviction that *the Master* was a great man and his works could be relied on. Will the growler do likewise? Not a bit of it!

